# iForal: Automated Handwritten Text Transcription for Historical Medieval Manuscripts

**DOI:** 10.3390/jimaging11020036

**Published:** 2025-01-25

**Authors:** Alexandre Matos, Pedro Almeida, Paulo L. Correia, Osvaldo Pacheco

**Affiliations:** 1 Instituto de Engenharia Eletrónica e Telemática de Aveiro (IEETA), Universidade de Aveiro, 3810-193 Aveiro, Portugal; pedro.bas@ua.pt (P.A.); orp@ua.pt (O.P.); 2 Instituto de Telecomunicações (IT), Instituto Superior Tecnico, Universidade de Lisboa, 1049-001 Lisbon, Portugal

**Keywords:** OCR, handwritten text recognition, text segmentation, automatic transcription, medieval manuscripts, Portuguese documents

## Abstract

The transcription of historical manuscripts aims at making our cultural heritage more accessible to experts and also to the larger public, but it is a challenging and time-intensive task. This paper contributes an automated solution for text layout recognition, segmentation, and recognition to speed up the transcription process of historical manuscripts. The focus is on transcribing Portuguese municipal documents from the Middle Ages in the context of the iForal project, including the contribution of an annotated dataset containing Portuguese medieval documents, notably a corpus of 67 Portuguese royal charter data. The proposed system can accurately identify document layouts, isolate the text, segment, and transcribe it. Results for the layout recognition model achieved 0.98 mAP@0.50 and 0.98 precision, while the text segmentation model achieved 0.91 mAP@0.50, detecting 95% of the lines. The text recognition model achieved 8.1% character error rate (CER) and 25.5% word error rate (WER) on the test set. These results can then be validated by palaeographers with less effort, contributing to achieving high-quality transcriptions faster. Moreover, the automatic models developed can be utilized as a basis for the creation of models that perform well for other historical handwriting styles, notably using transfer learning techniques. The contributed dataset has been made available on the HTR United catalogue, which includes training datasets to be used for automatic transcription or segmentation models. The models developed can be used, for instance, on the eSriptorium platform, which is used by a vast community of experts.

## 1. Introduction

The challenge of detecting and predicting patterns, letters, and forms within images has gained significant importance due to the rise of automatic recognition systems, which reduce the need for manual labor. Text recognition, particularly in printed documents, has advanced greatly, with Optical Character Recognition (OCR) now considered a solved problem. However, Handwritten Text Recognition (HTR), especially for historical documents, remains much more complex due to layout complexity, variable writing styles, and irregular spacing [1]. The automatic transcription of texts is an important area for various fields of knowledge and practical applications. It enables the digital storage, accessibility and dissemination of knowledge traditionally associated with physical media, such as paper, through the utilization of computer systems.

The manual transcription of these materials is a costly and time-consuming process [2]. The process is further complicated in the case of historical documents due to the degradation state of many documents and the necessity for specialists who are able to understand the type of writing used in these documents. Historical documents constitute a significant repository of knowledge about the past, cultures, societies, and influential individuals, their study being vital for interpreting and understanding our cultural heritage. For this reason, digitizing such documents and providing their transcriptions is important, both for future studies and for their preservation [3]. This reinforces the need to develop transcription systems as a way of accessing the texts contained in these documents.

**Figure 1:**
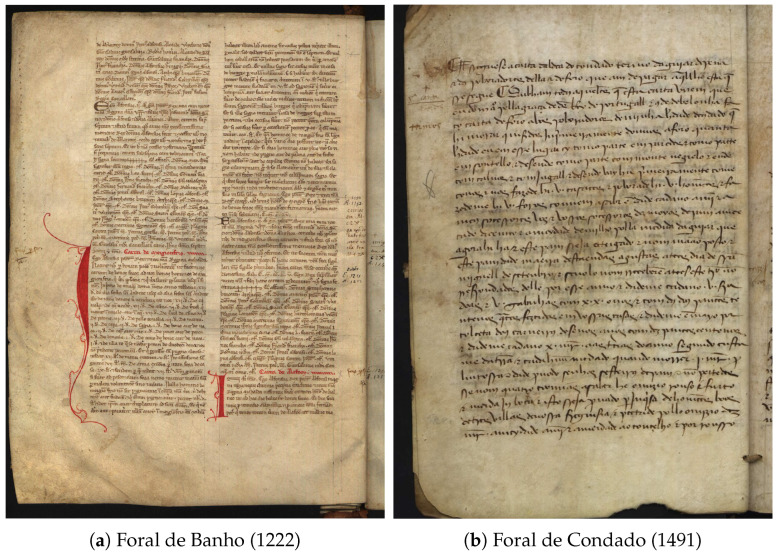
Examples of Portuguese medieval documents. Images obtained from the digital archive of Torre do Tombo: *digitarq.arquivos.pt*.

The present work is developed in the context of the iForal project [4,5], which has the objective of developing an electronic critical edition and glossary of Portuguese royal charters to facilitate research in the fields of history and linguistics. To achieve this objective, it is essential to obtain transcriptions of the texts contained in these manuscripts. The primary objective of this work is to develop a tool capable of automatically transcribing the textual content of digitized images of manuscripts, as illustrated in Figure 1. The creation of such a tool will facilitate the transcription process, reducing the time and effort required of paleographers. This work is concerned with Automatic Text Recognition (ATR), which encompasses three principal elements of the transcription process: layout recognition, text segmentation, and text recognition. The focus is on Portuguese medieval documents.

The processing of historical documents entails a series of stages to facilitate the transition of textual data from an image format to a digital one that computer systems can process, consisting of the following main steps: image acquisition, pre-processing, layout recognition, text segmentation, text recognition, and post-processing [3].

Depending on the solution adopted, not all systems follow all steps; however, these steps represent a general and complete pipeline of text recognition systems [1,6,7].

ATR systems can be divided into two types: OCR and HTR. HTR is a sub-area of OCR [8], where the focus is on recognizing handwriting, while OCR is more closely linked to recognizing printed text. These systems differ in the type of documents they are applied to, but both techniques fundamentally solve the same problem: recognizing text [3].

While HTR follows the same general process as OCR, it differs in its approach to layout recognition and text segmentation. These steps often result in higher error rates, especially for historical documents, as text layout can vary a lot due to irregular handwriting, making it difficult to accurately identify lines, words, and characters. For that reason, most of these systems rely on approaches based on line-level segmentation, called segmentation-free [1].

Segmentation-free techniques focus on recognizing all the characters in a segmented line [3]. The more frequently employed strategy in the state-of-the-art is the training of models using a Convolutional Recurrent Neural Network (CRNN) architecture [9,10], which is capable of recognizing text within segmented lines of text. However, for training purposes, this approach requires complete transcriptions of these lines, which in turn requires the input of paleography experts responsible for manually transcribing portions of the texts in order to create an initial training set [7]. Some studies, such as In Codice Ratio [2], have moved away from the segmentation-free paradigm and tried a segmentation-based approach. For In Codice Ratio, non-specialists were involved in the construction of the dataset with the objective of segmenting individual characters. Subsequently, a Convolutional Neural Network (CNN) was trained and employed to identify individual characters, thereby emphasizing the segmentation aspect in the HTR pipeline. Despite In Codice Ratio’s success, the number of individuals involved (120 students) may present a challenge for some transcription projects. More recently, new approaches are being investigated based on transformers, namely with the TrOCR architecture [11,12], showing great potential, even in the context of historical documents, but this architecture is not as widely used.

In the context of historical transcription projects, a number of tools have been developed with the objective of facilitating the use of these technologies and accelerating the transcription of documents. These tools are OCR engines and platforms. Engines are command-line tools that facilitate the development and application of models designed to streamline the transcription processes. Platforms are web applications designed for non-technical users, enabling the utilization of certain engines, and are employed for the management of transcription projects. Notable open-source tools include Kraken OCR [13], an engine that supports the development of both segmentation and recognition models, which is used by the eScriptorium platform [14], and Calamari OCR [15], an engine that focuses on the recognition step and is used by the OCR4all platform [1]. Another notable platform is Transkribus [16], which is not open-source but is widely used by academics interested in transcribing historical documents.

The main contributions of this paper are the following: (i) the creation of an annotated dataset, with information on text layout, line segmentation, and transcriptions of 67 Portuguese royal charters of the XIII, XIV, and XV centuries, made available to the research community (https://htr-united.github.io/share.html?uri=49ec62a02, accessed on 19 December 2024) for the training of machine learning models capable of detecting, segmenting, recognizing, and transcribing these texts; and (ii) a set of models for layout recognition, text segmentation, and text recognition, optimized for the text layouts and writing styles found in the above mentioned data.

In this work, layout recognition and text segmentation were performed using different techniques: YOLO [17], Kraken [13], and Mask R-CNN [18]. The recognition models presented here were developed using Kraken OCR and Calamari OCR, in order to facilitate their use and accessibility. This approach ensures that the trained models can be easily integrated into any transcription project, for instance, by using platforms such as eScriptorium or OCR4all.

In the remainder of this document, Section 2 presents the contributed dataset description and the methodology adopted for the text layout, segmentation, and recognition tasks. Section 3 presents the achieved results, which are discussed in Section 4. Section 5 presents conclusions and discusses directions for future work.

## 2. Materials and Methods

This section describes the proposed iForal-Dataset [19], as well as the proposed methodology for text layout recognition, text segmentation, and text recognition.

**Figure 2:**
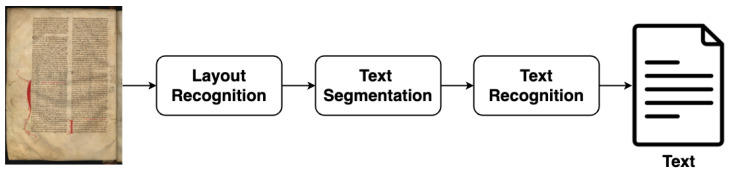
Generic architecture to transcribe a medieval manuscript.

Figure 2 represents the generic architecture used to transcribe medieval documents. The three main tasks involved are the following: (i) layout recognition, which detects the different elements of the document and extracts text blocks; (ii) text segmentation, which segments the detected text into lines; and (iii) text recognition, which transcribes the content of each line.

**Figure 3:**
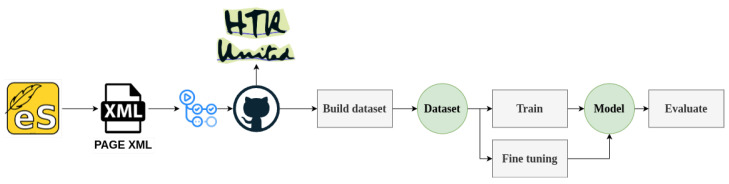
Representation of the adopted pipeline of actions.

Figure 3 illustrates the adopted procedure to take an image of a historical document and produce the corresponding transcription. The manuscripts were initially annotated using the eScriptorium platform, and the annotated documents were exported using the PAGE XML format [20], and stored in a GitHub repository (available at https://github.com/Arch-W/iForal-Dataset, accessed on 19 December 2024). After a series of GitHub Actions to ensure quality control of the data, to facilitate model training, and to enable inclusion of the resulting dataset in the HTR-United catalogue. The dataset was then used for training, fine-tuning, and the evaluation of the developed models.

### 2.1. iForal-Dataset

A new dataset, entitled iForal-Dataset, has been developed to facilitate the training and testing of HTR engines, and which includes metadata such as text regions, the position of the lines, and the respective transcription saved in the PAGE XML format [21]. To create the metadata, the eScriptorium framework [14] was used. The proposed dataset consists of 67 Portuguese historical documents, each containing between 1 and 12 pages and their respective transcriptions. These historical documents differ in their origin cities, dates (between the 13th and 15th centuries), and languages. Because each document only has a couple of text regions, 67 documents were a small sample to train a layout recognition model. For that reason, an extended dataset separated from the iForal-Dataset was also created to use in this task, using other Medieval documents (available at https://github.com/bastos-01/iforal-extended-dataset, accessed on 19 December 2024).

#### 2.1.1. Metadata Creation

As part of the iForal project, a platform [22] has already been developed that allows paleographers and academics, as well as the general public, to consult various Portuguese charters. The platform offers two types of transcription: paleographic and critical. Paleographic transcriptions reproduce source material faithfully. This type of transcription uses abbreviations and preserves writing errors and spelling mistakes, making it more challenging to read. The critical edition is meant to be easier to read, with expanded abbreviations and removed errors. Since the paleographic edition is the one that most faithfully replicates the textual content contained in the images, that was the type of transcription used to produce the metadata for inclusion in the proposed iForal-Dataset.

**Figure 4:**
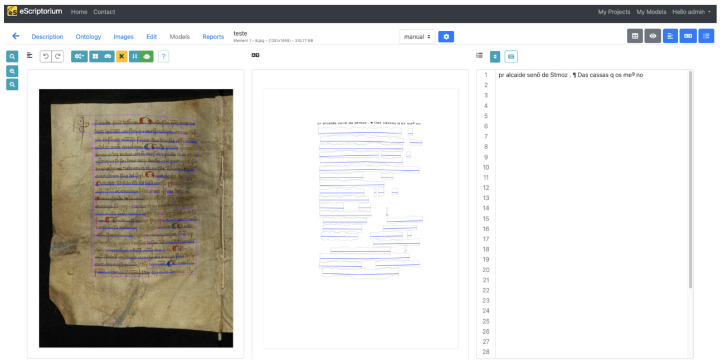
Example of an edited document in the eScriptorium platform.

Having access to the images of the manuscripts and their transcriptions, it is then possible to build the Ground Truth (GT), transforming the data into a format suitable for training and evaluating the automatic analysis models. As the models are trained based on images of lines of text, it was necessary to provide annotations for each of the lines contained in the charters. For this purpose, the eScriptorium platform was used, which enables the segmentation and linking of the lines within the image with the corresponding transcription, as illustrated in Figure 4. Finally, the metadata is exported in PAGE XML format [21].

#### 2.1.2. Normalization and Augmentation

The dataset revealed the presence of certain characters that seldom occur, which may imply that the machine learning models adopted are unable to learn their patterns and are therefore unable to recognize them. Consequently, some characters were normalized or converted to analogous versions. The normalization process involves the conversion of accented characters to their unaccented versions or the transformation of uppercase characters to their lowercase counterparts. The sole exception to this rule is the hyphen, which was transformed into a basic space due to the discovery that paleographers occasionally neglected to transcribe it in their transcriptions, resulting in the insertion of a space character instead. However, certain characters, despite having few occurrences, could not be normalized due to the fact that they were unique in their written form. Table 1 shows the normalizations made.

Furthermore, methods for enhancing the data were investigated. This process was designed to ascertain the extent to which increasing the dataset would result in improved outcomes. To this end, the dataset was augmented by applying a composition of transformations to 50% of the data.

The augmentation transformations applied include the following: (i) Randomly removing 5% of the pixels of the line image—this is applied with a probability of 20%. (ii) Subsequently, a distortion is applied to the image, organized into a grid of cells, with random displacements occurring at the intersection points of this grid. Following this distortion, one of three filters can be applied to blur the image. (iii) Finally, a novel type of distortion is applied, consisting of an elastic transformation of the manuscript text, resulting in slight deformation of the image of the line. When applying only the normalization process, a processed version of the dataset results, labelled as ‘Normalized’. A second version of the dataset considers the application of both the normalization and data augmentation, labelled as ‘Augmented’. Both datasets were used to test the recognition models, while for fine-tuning the recognition models, only the Normalized dataset was employed. The datasets were divided into three distinct subsets: 80% for training, 10% for validation, and 10% for testing.

### 2.2. Layout Recognition

Layout recognition and text segmentation are crucial components in HTR systems. Several approaches have been developed for this task, varying in complexity and scope. Some existing approaches focus on layout recognition, while others also consider text segmentation.

Pixel-level segmentation assigns labels to individual pixels, or to super-pixels, to identify document elements such as text blocks or images. Traditional methods, such as Support Vector Machines (SVMs), have been used to classify super-pixels, being computationally efficient. However, deep learning techniques like Fully Convolutional Networks (FCNs), particularly U-Net variants, offer improved accuracy by processing entire images in a single step, eliminating the need for pre- and post-processing [23]. The dhSegment model [24] extends this with additional steps, such as thresholding and morphological operations, to refine the results.

Instance-level segmentation treats layout recognition as an object recognition task. YOLOv5 has been applied to identify document layout elements [25], while more complex models combine object detection with segmentation masks to handle intricate layouts. Though effective, these methods can be computationally expensive [26].

After considering several alternatives, including different versions of YOLO and Kraken, the decision was to use YOLOv8, which can produce state-of-the-art results in object detection and segmentation across several computer vision problems.

YOLOv8, developed by Ultralytics [27], introduces architectural improvements over previous YOLO versions, including anchor-free detection, which reduces the number of possible predictions and speeds up training. The pre-processing steps include additional data augmentation techniques such as copy-paste and more advanced methods like CLAHE and image sharpening. YOLOv8 also uses adaptive augmentation techniques (AutoAugment, RandAugment), further enhancing performance during training. This version of YOLO aims to improve detection accuracy while reducing the training time, making it more efficient and robust for text block detection tasks.

Three tests were conducted with YOLO: the iForal-Dataset with 1 class (text block) and 3 classes (text block, image, capital), in this case using the extended dataset with 3 classes.

### 2.3. Text Segmentation

Text segmentation aims at the successful segmentation of lines, words, or characters to be provided to the recognition model.

Text line detection methods focus on extracting lines or words from document images. While earlier techniques typically combined LSTM and convolutional layers [18], modern models like Mask R-CNN dominate this space, offering better performance, especially for historical documents [18]. Mask R-CNN segments text by dividing images into overlapping patches and refining the results to avoid common errors.

Word and character-level segmentation are less commonly used due to high error rates, especially for dense, irregular handwriting, as typically found in historical documents. Some models attempt to segment words or characters based on pixel analysis. Still, these approaches often struggle with complex historical documents, leading to the adoption of more robust techniques. Segmenting at the line or word level leads to a more consistent input to the recognition models.

Since most modern recognition models work at the line level, further segmentation into words or characters is unnecessary. For this task, Mask R-CNN was selected, in line with the solutions reported in the state of the art.

The Mask R-CNN model [18], a state-of-the-art network, is designed for object detection and instance segmentation tasks. Builds upon the Faster R-CNN framework by adding a branch to predict segmentation masks for each detected object. The Mask R-CNN method is particularly well-suited for document line detection because it generates precise pixel-level masks, allowing for highly accurate segmentation. For pre-processing, the Mask R-CNN approach typically involves binarization to enhance the contrast between text and the background, image resizing, and normalization. Data augmentation techniques such as horizontal flipping, cropping, rotation, and noise addition can be applied, though their effectiveness varies depending on the specific implementation. The Mask R-CNN model combines a feature extraction backbone, typically a ResNet architecture (e.g., ResNet-50 or ResNet-101), with a feature pyramid network (FPN) and a region proposal network (RPN) [18]. These components work together to generate candidate regions and provide accurate and detailed line detection for documents.

### 2.4. Text Recognition

Regarding the recognition phase, the models developed were based on the most common approach in the state of the art, namely CRNN [9,10]. During the course of this work, a series of experiments were conducted, including the utilization of data augmentation techniques and the application of transfer learning for the training of new models. The goal is to assess the actual performance of the models in scenarios with limited data.

The models were developed utilizing two of the previously presented tools. The two tools in question are Kraken [13] and Calamari [28]. This will facilitate a direct comparison with existing state-of-the-art tools. Moreover, the creation of two models in these two tools enhances their accessibility and integration with other existing systems. By training models with Kraken or Calamari, they can be placed in a repository such as Zenodo, thereby enabling the wider community to access and utilize the models. As Kraken and Calamari serve as the foundational tools for the eScriptorium and OCR4all platforms, respectively, the models can be seamlessly integrated into any transcription project utilizing one of these platforms.

The models developed with Kraken can be classified into two categories: models trained with optimal parameters and models trained with transfer learning. Within each of these categories, there is the possibility of further subdivision into two additional categories: those models developed with a normalized dataset and those developed with an augmented dataset.

In order to identify the optimal parameters, a process of fine-tuning was conducted. Each model was trained for a total of 30 epochs, and the optimal parameters were selected based on a grid search method. In the case of Kraken, the parameters tested included the learning rate, batch size, scheduler, and augment (a Kraken parameter that applies data augmentation). While the range of values tested for each parameter was not exhaustive, it provides valuable insights into the performance of each combination. The learning rate was tested with two values: 0.001 and 0.0001. Similarly, the batch size was tested with two values: 1 and 4. The schedulers were tested with three values: constant, exponential, and reduction on plateau. The augment parameter was tested with two values: true and false. This was done to test the effect of using and not using the predefined data augmentation of Kraken.

Following the fine-tuning, more rigorous training sessions were conducted utilizing the optimal configuration of hyper-parameters. The training process employed both the original normalized data set and an augmented one. The maximum number of epochs was not imposed as a limitation, but early stopping was utilized with a lag of 20 epochs. This signifies that the training of the models ceased only if the performance of the validation metric did not enhance during 20 consecutive epochs.

In order to ascertain the extent to which a pre-trained model could be utilized, training was also carried out based on a pre-trained model on a dataset similar to Portuguese charters. The base model was trained on Old French and Latin manuscripts from the 8th to the 15th centuries [29]. The experiments conducted with this pre-trained model also focused on both the normalized and augmented datasets.

Similarly to Kraken, Calamari experiences were also generated in both datasets, namely normalized and augmented. However, in contrast to Kraken, Calamari’s experiences did not include training with a pre-trained model. Consequently, the only type of model trained was based on the optimal parameters identified in the fine-tuning process.

Once again, the technique employed during fine-tuning was grid search, with the objective of minimizing the CER for the validation set. This difference compared to Kraken is due to the fact that Calamari internally attempts to minimize this metric. The parameters tested were learning rate, batch size, and the network parameter, which is not a hyper-parameter but a configuration parameter of Calamari that modifies the underlying architecture. The values tested for these parameters were 0.001 and 0.0001 for the learning rate, 1 and 4 for the batch size and the three architectures already defined by default in Calamari, def, deep3 and htr+, were tested [28]. Furthermore, the training process of each model was constrained to 30 epochs, and all other parameters were set to their default values as defined by Calamari.

As with Kraken, once the optimal parameter values were identified, a more rigorous training process was initiated with Calamari. Two models were trained: one utilizing the normalized dataset and another based on the augmented one. To prevent overfitting, early stopping was employed with a lag of 20 epochs.

The processes described earlier led to two fine-tuning procedures for each tool, resulting in a total of six rigorous training processes utilizing early stopping on two datasets. Four of these are Kraken models, and two are Calamari models. These six models were subsequently compared, and conclusions were drawn to clarify the results obtained.

## 3. Results

The following sections present the results obtained for each HTR module.

### 3.1. Layout Recognition

Three tests were conducted to test the proposed layout recognition solution based on YOLOv8: (i) using the iForal-Dataset with one class (text blocks); (ii) using the iForal-Dataset with three classes (text blocks, images, capitals); and (iii) the extended dataset with three classes.

Table 2 represents the results for the layout recognition using the proposed YOLOv8 model, and also two other models: (i) YOLOv4, a previous version of the adopted model, to highlight the improvements possible after appropriately fine-tuning this new version, and (ii) the Kraken model, often present in the HTR platforms available. Notice that for Kraken, which extracts text lines from the detected regions for further recognition, the results are reported using different metrics. However, the mAP@0.50 metric was successfully implemented to better compare the various models.

‘Yv4’ and ‘Yv8’ represent versions 4 and 8 of YOLO, respectively. ‘1c’ and ‘3c’ determine if the model was trained with one labeled class (text block) or three labeled classes (text block, image, and capital). ‘ext’ refers to models trained with the extended dataset. The models starting with ‘K’ are trained with Kraken, from scratch and using transfer learning.

First of all, we can clearly see that the changes made in YOLO models improved their overall performance. Training with three classes enhanced every metric for both versions, especially in version 4. Extending the dataset was also the right decision, as almost every metric increased. With that in mind, version 8 of YOLO is undoubtedly the better-suited model for the layout recognition task, as its best training obtained metrics above 0.95. Although this is true, we can also conclude that the model with the initial dataset and three classes performs better in version 4. Essentially, version 4 has slightly better results with a smaller data sample to train, but version 8 really thrives with the larger dataset. Kraken’s region detection model also obtained very acceptable results. Even though its mAP@0.50 isn’t as high as in YOLO, its pixel accuracy and IoU are considerably higher than the others. This means that Kraken has more False Positives and False Negatives than YOLO, but the text box is much more accurate when detected. Overall, the best well-rounded and robust model is YOLOv8_3classes_extended, where YOLOv8 was trained with three labeled classes and the extended dataset. This is shown in almost every metric, where both mAP@0.50 and Precision-Recall obtained excellent values, indicating high accuracy without detecting almost any False Positives.

#### Visual Assessment

While the presented metrics effectively represent the model’s performance, visualizing the results can provide a clearer understanding of improvements and model comparisons. We chose to visually analyze only the best versions of each of the three models that achieved the best result (YOLOv4_3classes_ext, YOLOv8_3classes_ext, Kraken_reg_transfer).

In more straightforward cases, all models accurately detect text blocks, with YOLOv8 and Kraken regions being more precise. Documents featuring two columns are the most common among Portuguese documents. The main challenge arises with documents with uncommon layouts, where text and images appear in unusual positions.

**Figure 5:**
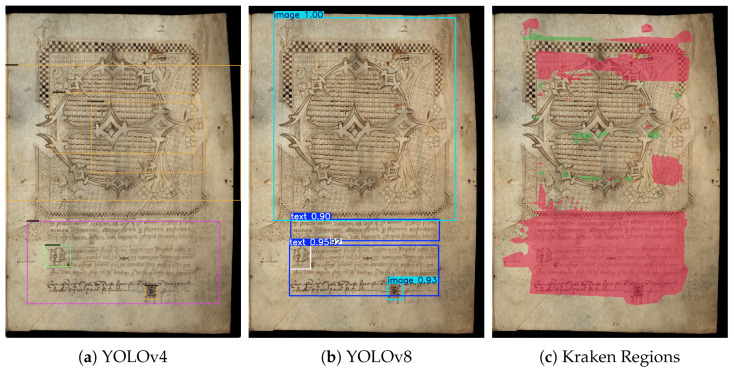
Layout recognition example.

Figure 5a,c show that YOLOv4 and Kraken detections were far from perfect, as the models struggle with complex and distinctive document layouts. Using YOLOv8 (Figure 5b) improves the performance in these cases, as it correctly detects the text blocks and has much fewer miss-detections.

### 3.2. Text Segmentation

Pixel accuracy can only be obtained in the Kraken network because Mask R-CNN works with masks (polygons) and not pixels. This is why additional metrics were included for both models, making it plausible to compare the results. As mentioned earlier, Kraken tests involve the default model, training from scratch, and transfer learning, while Mask R-CNN tests differ only in the number of epochs.

Table 3 describes the results for text segmentation models. Firstly, comparing each training in Kraken, the metrics improved significantly. As explained before, the default model is prepared to deal with historical documents and has very acceptable results. Therefore, it is expected that it will not register a huge improvement. Compared to the default model, the one trained using our dataset did not record a significant gain, which is expected, primarily because although it is trained with the same style documents, it uses much less data than the default model. This is why the default model was combined with the new dataset using transfer learning, allowing the robust default model to adapt to the Portuguese documents. In this training, we can see a significant improvement from the previous ones. The same pattern is noticeable regarding the Mask R-CNN. The number of epochs was incremented until there were no improvements and/or it started overfitting. Unfortunately, this network did not have a pre-trained model for text segmentation, so all the tests only used our dataset. Although the improvement is less significant than using transfer learning in Kraken, it performs better when trained with 75 epochs. After that, the performance starts to decrease. When comparing both networks in their best versions, they have very similar percentages of detected lines, showing that they both successfully detect about 95% of them. Mask R-CNN has a slightly higher mAP@0.50, meaning that it detects fewer false positives. The big difference is in the IoU values, where Kraken has a much lower value (0.669) when compared to Mask R-CNN(0.805). This means that even though they detect roughly the same number of lines, Mask R-CNN is much more accurate for each line. Overall, the best performer is undoubtedly Mask R-CNN_75, where it has the most lines detected with the most accuracy. This is visible in every metric, with a higher mAP@0.50 and a much higher IoU value, detecting the same amount of lines as Kraken.

#### Visual Assessment

Visualizing the results aids in comparing the models and their accuracy. However, it is only necessary to focus on the best-performing versions: Kraken_transfer and Mask R-CNN_75.

**Figure 6:**
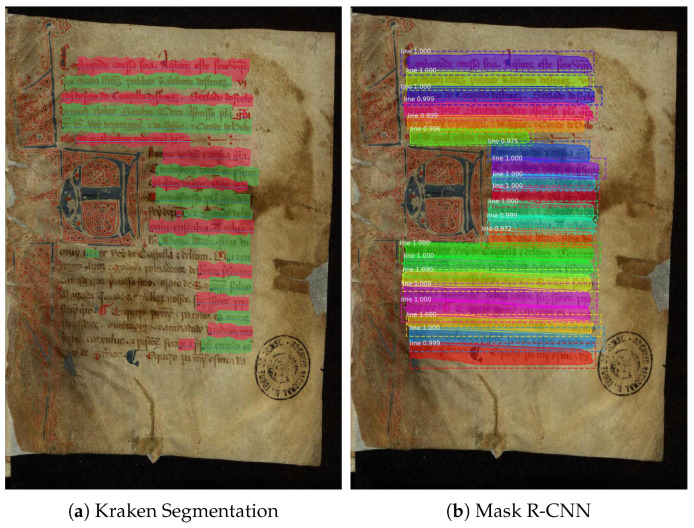
Text segmentation example.

Figure 6 demonstrates that Kraken’s performance decreases with less visible text compared to Mask R-CNN. Kraken misses the darkest portion of the text (bottom left corner), whereas Mask R-CNN detects it nearly perfectly.

### 3.3. Text Recognition

#### 3.3.1. Kraken Fine-Tuning

The Kraken fine-tuning process was completed using the configurations previously outlined in Section 2.4. This resulted in a total of 24 training sessions. The vast majority of combinations did not converge; however, the few that did converge yielded favorable results. Table 4 illustrates the combination of parameters that yielded the models with the best results. The table depicts the parameters employed to configure each training session, along with the results of CER and WER metrics and the time required for each training session.

It can be observed that all training sessions demonstrate a comparable level of performance in CER, with a mere 1% discrepancy between the highest and lowest recorded results. The same is true for WER, but the difference between the best and worst results is slightly more pronounced, around 3%.

As can be readily discerned from the table, all training sessions that utilize Kraken’s internal data augmentation option yield superior results in comparison to those that do not employ this option. This indicates that this parameter has a beneficial impact on the training process. The impact of data augmentation is more pronounced when we examine WER, with the results of the training sessions that utilize data augmentation falling between 30.41% and 31.23%, compared to the 32.30% to 33.65% range of the training sessions that do not utilize data augmentation.

The results also demonstrate that of the three parameters evaluated for schedule, only two, namely constant and reduce on plateau, are represented in the tables. This indicates that all training runs utilizing the exponential value failed to converge, thereby demonstrating that this value is unsuitable for training models with this dataset.

Similarly, of the two learning rates tested, 0.001 and 0.0001, only the value 0.0001 is included in the table. This further demonstrates that the training sessions using the 0.001 value did not converge, indicating that the value is too high.

Finally, a correlation between the ‘batch size’ and the ‘runtime’ is discernible. Training sessions utilizing a batch size of 4 exhibit a reduction in runtime of approximately 50% in comparison to those employing a batch size of 1. A comparison of the first and fourth results, which differ only in terms of the batch size, reveals a reduction in runtime from 6 h, 35 min and 39 s to 3 h, 22 min and 18 s. In terms of performance, CER increases from 10.07% to 10.26%, while WER increases from 30.41% to 31.23%. In both cases, the reduction in performance is marginal, although it is slightly higher for WER. In this instance, the larger batch size yields a marginal improvement in CER. However, when examining WER, the opposite is true, with the smaller batch size demonstrating superior performance.

The final four training sessions demonstrate that the larger batch size is slightly more effective than the batch size of 1 across all domains, both in terms of training duration and performance, when the augment parameter is not employed. Therefore, it can be concluded that although performance may occasionally decline slightly when utilizing a larger batch size, it considerably reduces training time, which may represent an optimal trade-off.

The best performance, in terms of both CER and WER, was achieved with the parameter combination in the first row of the table. Consequently, this combination was selected for prolonged training, extending beyond the 30 epochs used in fine-tuning. The results of these extended training sessions will be analyzed in Section 3.3.3.

#### 3.3.2. Calamari Fine-Tuning

The process of fine-tuning Calamari was completed in less time. This was due to the fact that one less parameter was used, resulting in a total of 12 training combinations. The Table 5 shows the results that achieved a CER of less than 20% in the validation set during fine-tuning. This table is sorted in descending order of the CER value, so that the first row of the table corresponds to the best parameter configuration found.

As with the models trained with Kraken, it is evident that all training sessions utilizing a batch size of 4 exhibited a reduction in training time. The reduction in training time is approximately 50% when compared to similar training sessions where only the batch size is changed to 1.

The learning rate that appears to yield the most optimal results is 0.001, as it is the value that is most prevalent in the table and for the most effective configuration. This is in contrast to the observations made with Kraken, where a value of 0.001 for the learning rate was insufficient to facilitate the convergence of the models.

The table also demonstrates that the network architecture with the optimal results was deep3. This architectural configuration ranks first and second, with 12.44% and 13.53% of CER, respectively, and fourth with 15.09% of CER. With regard to the other architectures, it can be seen that the standard Calamari architecture, def, obtained the poorest results of the three, as would be expected given that it is the simplest architecture. As for htr+, it appears only once, in third place, with a difference of approximately 2% when compared to deep3 for the same parameters. The parameters corresponding to the first line of the table were the best and were therefore used for more robust training sessions.

#### 3.3.3. Comparative Analysis

Table 6 presents a comparison of the CER and WER of the trained models, calculated on the test set. The best results obtained from both metrics are highlighted in bold. The table displays all the models that were obtained from the most rigorous training sessions. The name “Kraken-Best-Params” represents the models trained in Kraken using the best parameters found during fine-tuning, while “Kraken-Pretrained” represents the models trained in Kraken using the pre-trained model. Similarly, “Calamri-Best-Params” represents the models trained in Calamari using the best parameter configuration found during fine-tuning.

The best values obtained for both CER and WER belong to the model pre-trained on the normalized dataset. The model that was trained from scratch, with the optimal parameters identified through fine-tuning, exhibited a similar CER, but a slightly higher WER. The difference between the two models is marginal, and therefore it can be stated that both models perform identically. However, it is important to emphasize that the training times are different. In this case, the pre-trained model was able to converge more quickly, thus requiring less time. This indicates that the use of pre-trained models is a preferable strategy, as it reduces the time needed for training, while also obtaining excellent results.

The utilization of the augmented dataset appears to yield somewhat inferior outcomes when analyzing the models trained with Kraken, although there is a slight enhancement in the models trained with Calamari. This phenomenon can be attributed to the augmentation parameter employed in all models trained with Kraken. The act of augmenting already augmented data can result in a reduction in image quality to a degree that affects the models’ performance to a slight extent.

Finally, it is evident that the models trained with Calamari exhibit inferior performance compared to those trained with Kraken. This discrepancy may be attributed to the differing underlying architectures of the two tools.

### 3.4. Visual Assessment

This subsection presents a selection of transcriptions generated by the optimal model. These examples are provided to illustrate the quality of the model’s output. They encompass both optimal results and instances where the transcriptions are of inferior quality. All examples are drawn from the test set of the dataset.

**Figure 7:**
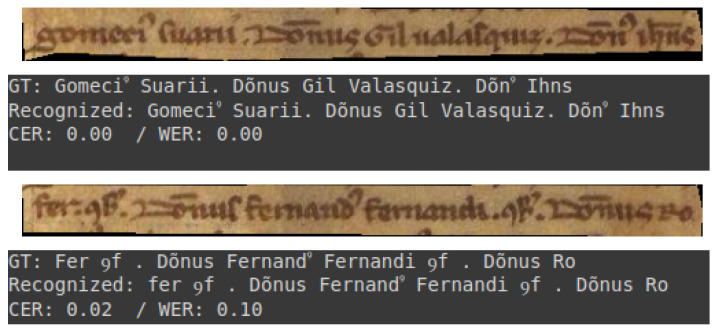
Reliable transcription examples.

**Figure 8:**
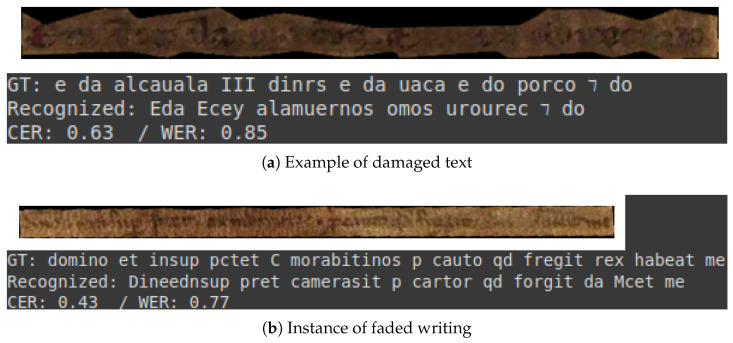
Flawed transcription examples.

The initial two examples, depicted in Figure 7, exemplify a commendable level of transcription. Conversely, Figure 8a,b illustrate instances of transcription that deviate from this standard, which can be attributed to the intrinsic characteristics of the documents in question.

It is evident that the model is capable of performing transcriptions that require minimal or no correction, as illustrated in Figure 7. However, in cases where documents are damaged, as illustrated in Figure 8, the model’s performance drops considerably, necessitating more human intervention.

## 4. Discussion

The effort to train and apply a machine learning network for detecting and segmenting lines of text in Portuguese historical documents was successfully completed, yielding improved performance compared to state-of-the-art models for Portuguese documents. Three key tasks were required: layout recognition, text segmentation, and text recognition.

For layout recognition, both YOLO versions and the Kraken regions model outperformed the default models, with YOLOv8 standing out when trained on the extended dataset using three classes.

Text segmentation achieved impressive results, with Kraken and Mask R-CNN performing effectively. Although transfer learning enhanced Kraken’s performance, Mask R-CNN trained over 75 epochs emerged as the better option, detecting the same number of lines with higher accuracy. Considering the complexity of the layouts and writing styles in each document, these outcomes can be regarded as highly positive.

Although the results are highly positive, there is still potential for further enhancement. A significant limitation is the dataset, which relies on external assistance to transcribe documents into digital text (from the [4] project). As a result, only transcribed documents are usable, restricting the dataset size. It is anticipated that results will improve with access to a larger dataset. While most state-of-the-art methods focus on line-level segmentation, word- and character-level segmentation could also be explored. Comparing these methods to the approaches used in this paper could provide valuable insights.

For text recognition, the best results were obtained by the pre-trained model in Kraken with the normalized dataset. This model achieved a CER of 8.1% and a WER of 25.5%, thus proving capable of recognizing these Portuguese documents. The best model trained with Calamari also shows positive results, although lower than the best model. This model achieved a performance of 12.4% CER and 36.1% WER on the same dataset.

The text recognition results show a significant discrepancy between the performance metrics. While the CER is close to or even less than 10%, the WER does not even reach 20%. The low CER indicates a high degree of similarity between the text recognized by the model and the GT. Nevertheless, the elevated WER suggests that the errors are distributed across multiple words. In some instances, this can be attributed to a single character being misidentified, which results in the incorrect identification of words.

This suggests that better results can still be achieved. Therefore, in order to further improve the results obtained, we suggest training and applying a Language Model (LM) to the results returned by the recognition models [30]. The application of an LM in a post-processing stage of the results obtained from the recognition models is expected to improve not only the WER but also the CER.

Another area for future work is training models based on the TrOCR architecture. As a relatively recent transformer-based architecture, it is not yet widely adopted. However, as demonstrated in some studies [12], it shows significant potential for performance improvements. For these reasons, exploring this alternative in future research will be worthwhile.

## 5. Conclusions

Effective layout recognition and text segmentation models were developed to detect, extract, and segment text from Portuguese medieval documents. These models demonstrated good performance in breaking text down into lines, enabling the recognition model to process them efficiently.

The conclusion of this research also led to the creation of recognition models that are capable of recognizing texts from Portuguese charters with a very satisfactory level of performance. These models can be used within the tools for which they were created, but they can also be integrated into two open-source platforms, OCR4all and eScriptorium. Their integration into these platforms allows them to assist palaeographers in transcribing new documents in a more user-friendly way.

Furthermore, the provision of these models facilitates the training of new models, as they can be employed as a foundation for the development of more resilient models. This allows new models to be trained on similar datasets more quickly and with better results.

The provision of the models thus contributes in two ways to advances in this field. Firstly, it facilitates the transcription of existing documents that have not yet been transcribed. Secondly, it enables the training or refinement of new models on similar corpora.

Finally, another significant contribution was the creation and publication of the GT used to train the models. It is expected that this Portuguese charter dataset will subsequently be employed to facilitate the training of new models.

## Figures and Tables

**Table 1:** Character normalizations considered.

Original	á	ñ	í	Ã	Ĩ	$\hat{j}$	-	K	U	Z	Ç	J	U+2019	U+A76E
Normalized	a	n	i	ã	ĩ	j	SPACE	k	u	z	ç	j	U+0027	U+A76F

**Table 2:** Layout recognition results.

Model	mAp@0.50	Precision	Recall	f1-Score	IoU	Pixel Acc.
Yv4_1c	0.77	0.88	0.74	0.80	0.70	-
Yv4_3c	0.91	0.91	0.78	0.84	0.78	-
Yv4_3c_ext	0.93	0.89	0.89	0.89	0.77	-
K_reg_scratch	0.84	-	-	-	0.92	**0.98**
K_reg_transfer	0.88	-	-	-	**0.95**	**0.98**
Yv8_1c	0.85	0.82	0.85	0.84	-	-
Yv8_3c	0.87	0.91	0.89	0.85	-	-
**Yv8_3c_ext**	**0.98**	**0.98**	**0.95**	**0.96**	-	-

**Table 3:** Text Segmentation results.

Model	mAP@0.50	% Lines Detected	IoU	Pixel Acc.
Kraken_default	0.821	94.1%	0.612	0.971
Kraken_scratch	0.843	94.2%	0.620	**0.976**
Kraken_transfer	0.878	**95.1%**	0.669	0.975
MaskRCNN_30	0.897	94.0%	0.797	-
MaskRCNN_50	0.899	94.0%	0.804	-
**MaskRCNN_75**	**0.908**	**95.1%**	0.805	-
MaskRCNN_100	0.896	94.1%	**0.814**	-

**Table 4:** Kraken fine-tuning best results.

Runtime	Augment	Batch Size	L. Rate	Schedule	CER	WER
6:35:39	TRUE	1	0.0001	constant	**0.1007**	**0.3041**
3:22:48	TRUE	4	0.0001	reduceonplateau	0.1019	0.3116
6:35:21	TRUE	1	0.0001	reduceonplateau	0.1022	0.3086
3:22:18	TRUE	4	0.0001	constant	0.1026	0.3123
3:22:41	FALSE	4	0.0001	reduceonplateau	0.1073	0.3230
3:21:45	FALSE	4	0.0001	constant	0.1073	0.3226
6:48:00	FALSE	1	0.0001	constant	0.1094	0.3241
6:47:35	FALSE	1	0.0001	reduceonplateau	0.1108	0.3365

**Table 5:** Calamari fine-tuning best results.

Runtime	Batch Size	Network	Learning Rate	CER
01:51:18	4	deep3	0.001	**0.1244**
07:05:24	1	deep3	0.0001	0.1353
01:50:56	4	htr+	0.001	0.1458
03:17:25	4	deep3	0.0001	0.1509
02:18:49	1	def	0.001	0.1817
01:00:27	4	def	0.001	0.1911

**Table 6:** Model comparisons.

Model	Dataset	CER	WER
Kraken-Best-Params	Normalized	**0.081**	0.256
Kraken-Best-Params	Augmented	0.087	0.269
Kraken-Pretrained	Normalized	**0.081**	**0.255**
Kraken-Pretrained	Augmented	0.087	0.263
Calamri-Best-Params	Normalized	0.129	0.373
Calamri-Best-Params	Augmented	0.124	0.361

## Data Availability

The dataset used in this work can be found in the HTR-United catalogue and can be accessed at https://htr-united.github.io/share.html?uri=49ec62a02, accessed on 19 December 2024.
